# Ninjin’yoeito Kampo medicine enhances zebrafish endurance under forced-swimming conditions via muscle hypertrophy

**DOI:** 10.1007/s11418-026-02020-x

**Published:** 2026-04-21

**Authors:** Kaita Hirano, Jihwan Eum, Haruri Nagata, Yuko Kamada-Futagami, Toshiki Hyodo, Momoko Kawabe, Seiwa Michihara, Shigeki Chiba, Akio Inui, Kazuhiro Shiozaki

**Affiliations:** 1 Department of Food Life Sciences, Faculty of Fisheries, Kagoshima University, Kagoshima, Japan; 2 Course of Biological Science and Technology, The United Graduate School of Agricultural Sciences, Kagoshima University, Kagoshima, Japan; 3 Kampo Research Laboratories, Kracie Ltd., Toyama, Japan; 4 Pharmacological Department of Herbal Medicine, Graduate School of Medical and Dental Sciences, Kagoshima University, Kagoshima, Japan

**Keywords:** Fast muscle, Kampo medicine, Muscle hypertrophy, Ninjin’yoeito, Zebrafish

## Abstract

Maintaining skeletal muscle mass and function is essential for extending healthy life expectancy in aging populations. While exercise and nutritional support are primary strategies to counteract sarcopenia and frailty, their effectiveness is often limited in the elderly. Thus, pharmacological and dietary interventions that directly enhance muscle formation have attracted increasing attention. Ninjin’yoeito (NYT), a traditional Japanese Kampo medicine composed of 12 medicinal herbs, has recently been highlighted for its antifrailty potential. In this study, we examined the effects of NYT dietary supplementation on muscle hypertrophy and endurance in zebrafish. NYT administration did not alter food intake or spontaneous locomotion under standard conditions, but it markedly improved endurance performance in a forced swimming test. NYT-fed zebrafish displayed prolonged upstream swimming times and delayed fatigue onset. Histological analysis revealed hypertrophy of fast-twitch muscle fibers, and gene expression profiling demonstrated upregulation of *myhz2*, *actn3*, and *myod*, which are key regulators of fast muscle development and differentiation. In contrast, genes associated with slow-twitch fibers and canonical protein synthesis pathways were unaffected. Furthermore, *ldh*, a pivotal enzyme in anaerobic glycolysis, was significantly upregulated in NYT-treated fish following exercise, suggesting enhanced energy metabolism. Collectively, these findings indicate that NYT promotes fast-twitch muscle hypertrophy and enhances physical performance, supporting its potential as a functional intervention to prevent or attenuate age-related decline in muscle health.

**Graphical abstract:**

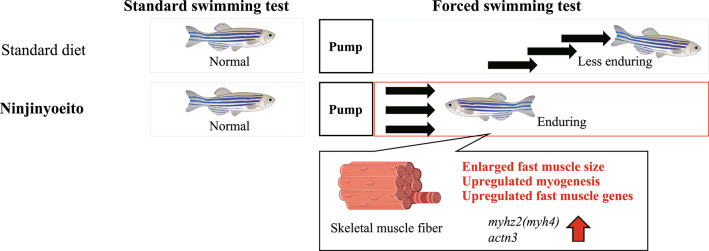

**Supplementary Information:**

The online version contains supplementary material available at 10.1007/s11418-026-02020-x.

## Introduction

Life expectancy has been steadily increasing worldwide, with the average lifespan in Japan reaching approximately 81.1 years for men and 87.1 years for women in 2016 [1]. However, the average healthy life expectancy—defined as the period during which individuals can live independently without substantial health-related limitations—remains considerably shorter, at around 72.6 years for men and 76.9 years for women. This gap highlights the importance of strategies that not only extend lifespan but also preserve physical capacity and quality of life. Skeletal muscle health is a critical determinant in this context, as muscle mass and function are closely associated with mobility, energy metabolism, and resistance to frailty.

Frailty and sarcopenia, both major public health concerns in aging societies, are typically characterized by the progressive loss of skeletal muscle mass and strength. While muscle atrophy prevention is crucial, accumulating evidence indicates that promoting skeletal muscle hypertrophy—an increase in muscle fiber size and contractile strength—represents an equally important therapeutic goal for extending healthspan. Regular physical exercise is a well-established approach for maintaining muscle function [2], yet adherence can be difficult in elderly populations due to physical limitations or comorbidities. Consequently, pharmacological interventions and functional foods capable of directly enhancing muscle growth and endurance performance have attracted increasing attention.

Numerous naturally derived compounds have been investigated for their potential to preserve muscle mass. Ursolic acid, isoflavones, and tomatidine suppress muscle atrophy in animal models and cultured cells [3–5]. Beyond anti-atrophic effects, plant-derived agents with direct anabolic activity have also garnered attention. Ecdysteroids, for instance, exert anabolic effects via estrogen receptor binding and have been shown to increase muscle fiber size in rodents, leading to their marketing as dietary supplements for enhancing recovery, strength, and fatigue resistance [6]. These findings underscore the potential of phytochemicals not only as protective agents against muscle wasting but also as proactive promoters of muscle hypertrophy.

Kampo medicine, a traditional Japanese herbal system derived from Chinese medicine, consists of multiple botanical and mineral components with complex pharmacological actions. Because of their multicomponent nature, Kampo formulations may exert synergistic effects and are often considered safer for long-term administration compared to synthetic drugs [7]. These properties make Kampo medicines particularly attractive for elderly patients requiring multifaceted support. Among them, Ninjin’yoeito (NYT)—a representative formula comprising 12 medicinal herbs including Rehmannia root, ginseng, cinnamon bark, and Schisandra fruit—has been widely studied for its antifrailty potential. Traditionally, NYT has been indicated for constitutional weakness and “qi- and blood-deficiency” patterns and has been clinically used for fatigue recovery and convalescence in modern Japanese practice, including symptoms such as fatigue, anorexia, anemia, and cold intolerance [8, 9]. NYT has been reported to exert anxiolytic and prosocial effects that may alleviate psychological and social dysfunction [10, 11], prevent fast-twitch fiber atrophy in klotho-hypomorphic mice through suppression of Atrogin-1 and Murf1 expression [12], and alleviate fatigue-like behaviors in senescence-accelerated mice [13]. These traditional indications, together with emerging preclinical evidence, suggest that NYT may support physical functioning through muscle-centric mechanisms. However, whether NYT can directly induce skeletal muscle hypertrophy independent of atrophy prevention remains unresolved. Clarifying this point would provide novel insights into its application as an antifrailty intervention.

Zebrafish (*Danio rerio*) are small teleost fish that have become powerful vertebrate models for studying human physiology and disease, including muscle biology. Particularly in recent years, the importance of zebrafish in aging research has been increasingly recognized, further supporting their utility in studies of muscle physiology and healthy lifespan extension [14, 15]. Because they swim continuously, zebrafish naturally serve as exercise models [16], and the molecular pathways regulating hypertrophy are highly conserved with those of mammals [17]. Their suitability for high-throughput screening also makes them valuable tools for identifying functional food ingredients that promote muscle growth. Indeed, compounds such as ferulic acid have been shown to induce hypertrophic muscle growth in zebrafish [18]. Building on these advantages, the present study investigated the effects of NYT dietary supplementation on muscle hypertrophy and exercise performance in young adult zebrafish under non-atrophic conditions, aiming to establish its role as a functional intervention for promoting muscle development and extending healthy aging.

## Materials and methods

### Fish

Young adult zebrafish were purchased from a local aquarium supplier in Kagoshima City, Japan. The fish were housed in 5 L tanks under controlled environmental conditions with a photoperiod of 14 h light and 10 h dark. The animals were fed a commercial diet (Otohime B2; Marubeni Nisshin Feed Co., Tokyo, Japan) twice daily. All animal experiments were performed in accordance with ethical guidelines and regulations approved by the Animal Care and Use Committee of Kagoshima University.

### Feeding experiment

Feeding trials were conducted in 2 L tanks filled with water maintained at 26 °C. Prior to the experiment, three-month-old zebrafish (average body weight: 0.2 g) were acclimated to a standard commercial diet. In the present study, the fish were used at a young-adult stage, and because of their small body size, males and females were not distinguished and were tested in mixed groups. NYT (Lot No. 16033006) was provided as a freeze-dried hot-water extract by Kampo Research Laboratories (Kracie Ltd., Toyama, Japan). All herbs comprising NYT were listed in Table 1. Each plant material was identified based on its external morphology and authenticated by marker compounds in accordance with the methods described in the Japanese Pharmacopoeia and our in-house standards. The evaluation of quality control using HPLC and extraction yield of NYT were detailed in our previous report [11]. NYT was incorporated into the experimental diets at a concentration of 0.3 and 3% (w/w), based on previous protocols established in zebrafish studies [11, 19]. These concentrations were selected based on previous findings [10, 11, 19]. The control diet (Cntr) was prepared identically, excluding NYT. At this concentration (3% NYT), the estimated daily intake of NYT in zebrafish corresponded to approximately tenfold the clinical human dose based on body weight [19].

**Table 1:** Formulas of Ninjinyoeito (NYT)

Component herbs	Weight (g)
	NYT^1^
Rehmannia root	4
Japanese Angelica root	4
Atractylodes Rhizome	4
Poria Sclerotium	4
Ginseng	3
Cinnamon Bark	2.5
Polygala root	2
Peony root	2
Citrus Unshiu Peel	2
Astragalus root	1.5
Glycyrrhiza	1
Schisandra Fruit	1

^1^Amount of herbs for the preparation of 6.7 g NYT extract

**Fig. 1:**
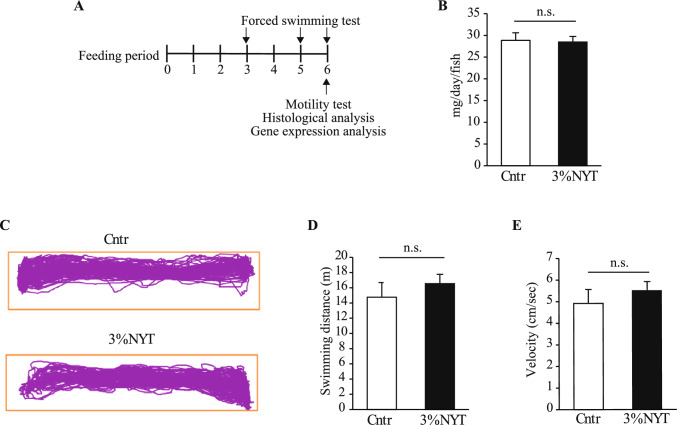
Effects of 3% NYT on feeding behavior and spontaneous locomotor activity in zebrafish. **A** Experimental timeline of dietary NYT administration and behavioral assessments. **B** Average of daily food intake for six weeks in 3% NYT-fed and control groups. **C**–**E** Spontaneous swimming behavior was analyzed after acclimation to a standard tank. **C** Swimming trajectory. **D** Total distance traveled. **E** Average velocity. n = 6. n.s., not significant

Fish were randomly assigned to either the NYT group or control group and fed their respective diets to apparent satiation twice daily for a duration of 7 weeks (Fig. 1A). To ensure that no feed remained uneaten, the diets were provided in small portions incrementally until feeding ceased [20]. The total amount of feed supplied to each tank was weighed daily, and food intake was calculated as the average daily feed amount per fish by normalizing to the number of fish in each tank. Forced swimming tests were performed at weeks 3, 5, and 6 to assess the endurance capacity. During the final week, spontaneous swimming behavior was recorded under standard tank conditions. Muscle samples were collected for histological analysis at week 6, and gene expression analysis was conducted following the last forced swimming test.

No widely accepted positive control compound for inducing skeletal muscle hypertrophy in zebrafish under non-atrophic conditions has been established to date. However, previous studies in mammalian models, including mice, have demonstrated that ginseng (Gin) promotes skeletal muscle hypertrophy and enhances muscle function [21]. Therefore, in the present study, Gin extract (Lot no. T160623, Kracie Ltd.) was used as a reference extract. A Gin-containing diet was prepared so that the estimated intake corresponded to 50 μg of Gin extract per fish per day, a dose selected with reference to dosing regimens commonly used in rodent studies [22]. Accordingly, a diet containing 0.25% (w/w) ginseng extract was formulated, assuming a daily feed intake of approximately 20 mg per fish. Fish were fed this diet under the same experimental conditions as the NYT and control groups.

### Evaluation of spontaneous locomotion in a standard tank

Spontaneous swimming behavior was assessed using a standard tank assay as previously described [23]. Briefly, individual zebrafish were transferred to a transparent observation tank (21 cm × 5 cm × 17 cm) and allowed to acclimate for 30 min. After acclimation, swimming behavior was recorded for 5 min using a video camera. The camera was positioned directly above the tank. Behavioral parameters, including swimming trajectory, total distance traveled, and average velocity, were analyzed using the ANY-maze behavioral tracking software (Stoelting Co, IL, USA).

### Forced swimming test in a flow tunnel

**Fig. 2:**
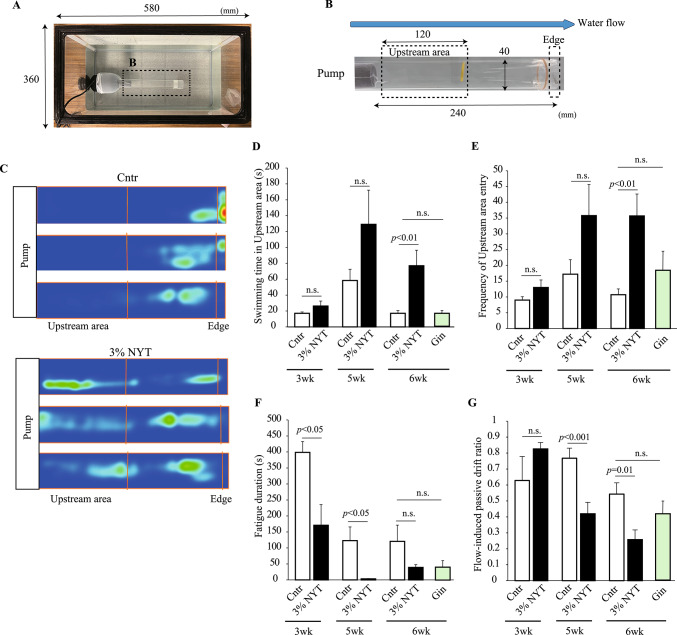
Effects of 3% NYT on swimming performance under forced swimming conditions. **A**, **B** Schematic of the forced swimming test apparatus and tunnel configuration. **C** Representative heatmaps of swimming trajectories in control and 3% NYT-fed zebrafish at week 6. Color intensity indicates the density of swimming activity, with warmer colors (red to yellow) representing higher occupancy and cooler colors (green to blue) indicating lower occupancy within the tunnel during the observation period. **D**–**G** Quantitative analysis of swimming performance at weeks 3, 5, and 6. **D** Time spent in the upstream area. **E** Frequency of upstream entries. **F** Fatigue duration. **G** Flow-induced passive drift ratio. Gin, ginseng containing diet. n = 6–10

To evaluate swimming performance under physical stress, a forced swimming test was conducted according to a previous report [24] with modifications using a 70 L tank system (Fig. 2A). A custom-built acrylic tunnel (4.0 cm inner diameter × 24 cm length) was installed inside the tank, with one end connected to a submersible water pump (SLW-30, Jebao, Guangdong, China) and the opposite end fitted with a mesh screen (Fig. 2B). The tunnel was conceptually divided into upstream and downstream areas. Each fish was individually introduced into the tunnel. The water flow was initially set to 0.065 m/s and maintained for 30 s for acclimation, followed by a stepwise increase to 0.075 m/s and 0.080 m/s, each with a 30-s acclimation period. Finally, the flow rate was adjusted to 0.090 m/s, and behavioral analysis was conducted for 10 min. The camera was positioned at the side of the tank. This maximum velocity was selected based on preliminary experiments demonstrating that zebrafish can sustain swimming at this velocity for a short duration without being immediately swept downstream. The duration spent in the upstream area and the frequency of upstream area entries were measured as indices of swimming endurance. When a fish was carried downstream to the mesh end, gentle tapping of the outer acrylic wall (not the fish directly) was used to encourage the resumption of swimming. The time spent resting on the mesh was recorded as fatigue duration. Additionally, a “Flow-induced passive drift” was defined as a rapid downstream displacement in which the fish was swept to within 3 cm of the tunnel terminus in a single motion. The flow-induced passive drift ratio was calculated as a measure of the resistance to downstream flow, using the following equation:

$$\begin{aligned}&{\text{Flow-induced passive drift ratio}}\, = \,{\text{frequency of upstream entries}}\\ & \quad / {\text{frequency of Flow-induced passive drift reaching the tunnel end}}.\end{aligned}$$

### Immunohistochemical analysis of muscle tissue

Zebrafish were euthanized under anesthesia with 0.1% MS-222, and their bodies were immediately dissected. Heads and tails were removed using a scalpel, and the remaining trunk regions were embedded in Optimal Cutting Temperature (OCT) compound and rapidly frozen in a dry ice/hexane mixture at − 60 °C. Cryosections were prepared at a thickness of 20 µm using a cryostat maintained at − 20 °C. Sections were mounted on glass slides, air-dried, and fixed in 4% paraformaldehyde for 10 min at room temperature. After rinsing in phosphate-buffered saline, the sections were blocked with goat serum and incubated overnight at 4 °C with a rabbit polyclonal anti-laminin antibody (1:100 dilution, GTX113016, GeneTex, CA, USA). After washing, sections were incubated with a fluorescence-conjugated secondary antibody. Fluorescent signals were visualized using a fluorescence microscope equipped with an Apotome module (Carl Zeiss, Oberkochen, Germany). The cross-sectional areas of the muscle fibers in the fast-twitch region were quantified based on laminin staining using ImageJ software (NIH, MD, USA).

### Quantitative real-time PCR

The skeletal muscle was dissected 10 min after completion of the forced swimming test. Total RNA was extracted from zebrafish muscle tissue using Sepasol-RNA I Super G (Nacalai Tesque, Kyoto, Japan) according to the manufacturer’s protocol. First-strand cDNA was synthesized from 0.5 µg of total RNA using the ReverTra Ace qPCR RT Master Mix with gDNA Remover (TOYOBO, Tokyo, Japan). Quantitative real-time PCR was performed on a StepOne Real-Time PCR System (Thermo Fisher Scientific, Waltham, MA, USA) using the KOD SYBR Green PCR Master Mix (TOYOBO). Used primers were listed in Table 2. Relative gene expression levels were calculated by normalization to *actb* (β-Actin) mRNA expression.

**Table 2:** Primers used in this study

Gene	Primers
*actb*	5′-CGCCATACAGAGCAGAAGCCA-3′
	5′-AGCACCCTGTGCTGCTCACT-3′
*myod*	5′-AGCAAGGTCAACGACGCTTT-3′
	5′-GCTGTTCCGTCTTCTCGTCTG-3′
*myog*	5′-AGGCCGCTACCTTGAGAGA-3′
	5′-CACTAGAGGACGACACCCCA-3′
*myh1*	5′-CTTATGCGAAGTCTGAGGCAC-3′
	5′-AGGTTGTCTTGCTCCGCTTG-3′
*myhz2*	5′-TCAAGGAACGCAAGTAAGCC-3′
	5′-CTTCTCAGGCTTACGGAGGA-3′
*myh7*	5′-CTTGGTGCACATCAGACAAGG
	5′-GGTAAGAAGCTGCTGCTCCAA-3′
*actn3*	5′-GAGAATCACTGGAACGTGTGG-3′
	5′-TGCACAATGAACATGTCCTGC-3′
*pgc1a*	5′-AGTACCGACGCGACTATGAG-3′
	5′-ACTCGCCTCTCCTCATTGC-3′
*pparb*	5′-CCGTCACCGAACTGACTGAA-3′
	5′-GCCTCATGGACACCGTACTT-3′
*s6k*	5′-TGCAGTTGGAGACGGAGAATG-3′
	5′-CTCGAACTGCGAAGCATCT-3′
*4ebp1*	5′-AAGACCACCAGTCAGGCAAT-3′
	5′-TGTCGAGTAATCATGAGGCAGA-3′
*gpx*	5′-CCCTCTGTTTGCGTTCCTGA-3′
	5′-TCTTGAATGGTTCCCCGTCC-3′
*cat*	5′-CTCCTGATGTGGCCCGATAC-3′
	5′-TCAGATGCCCGGCCATATTC-3′
*il6*	5′-TCAACTTCTCCAGCGTGATG-3′
	5′-TCTTTCCCTCTTTTCCTCCTG-3′
*mct4*	5′-CAAATGGTGTGGCCGTAGAG-3′
	5′-CACCACAACATCAAACACATCC-3′
*cs*	5′-TATTGCACGCTTGCTCTCAG-3′
	5′-ATGGAGTGCTTATGGAGCGA-3′
*pfk*	5′-CTGTGTAATCGGCGGTGATG-3′
	5′-TTGGCCTCTTCCTTGGTGAT-3′
*ldh*	5′-GAACCGTGTGATTGGCAGTG-3′
	5′-CTCCATGTTCTCCAACGACC-3′
*mafaa*	5′-GTCTCGTTGCTCTAAGGACG-3′
	5′-TACTCGTCCTCACTTCGCTG-3′
*mafba*	5′-CTCAAGCAGGAGATCAACCG-3′
	5′-CACGCACTCACATGAAGAACT-3′
*mafbb*	5′-TGCCAGAGCAATAATGGACC-3′
	5′-TTCAACTGGACAGACCAGCAA-3′
*maff*	5′-CTGAACACAGCCTCCGTCAT-3′
	5′-GGCGATGTTCTAGGATGCTC-3′
*mstnb*	5′-CCCGTTAAGGACGGAGGAAG-3′
	5′-CCAGCCAAAGTCCTCGAAGT-3′

### Data analysis

All data are presented as mean ± standard error of the mean (SEM). Statistical comparisons between the two groups were performed using unpaired two-tailed Student’s t-test. Statistical significance was set at p < 0.05. For comparisons between three groups, values were analyzed using one-way analysis of variance (ANOVA), followed by Tukey’s multiple comparisons test.

## Results

### Effects of NYT on baseline locomotor activity and feeding behavior

To evaluate the baseline effects of NYT on zebrafish behavior, we first examined food intake and spontaneous swimming activity under non-stressed conditions. Daily food consumption did not differ significantly between the control group and the groups fed NYT at 3% (Fig. 1B), indicating that NYT supplementation did not affect appetite.

Next, we assessed spontaneous locomotor activity using a standard tank assay. The zebrafish were transferred to the test tank and allowed to acclimate sufficiently prior to behavioral assessment because NYT has previously been shown to exert anxiolytic effects [10] that may influence zebrafish behavior. Following acclimation, swimming trajectories, total distance traveled, and average velocity were analyzed. No significant differences were observed between the NYT and control groups in any of these parameters (Fig. 1C–E), suggesting that NYT did not alter basal swimming behavior in zebrafish.

### Effects of NYT on forced swimming performance

To assess the effects of NYT on swimming endurance under physical stress, we conducted a forced swimming test using a flow tunnel system. Zebrafish were exposed to increasing water flow over a 10 min period, and their swimming behavior was recorded (Fig. 2A, B). In addition to the NYT group, we also tested zebrafish fed a Gin-supplemented diet as a reference condition. Representative heatmaps showed that at week 6, the control fish were frequently pushed downstream and accumulated near the mesh screen at the end of the tunnel, whereas zebrafish fed the 3% NYT diet tended to remain in the upstream area near the pump (Fig. 2C).

To quantify this behavioral difference, we evaluated four parameters: the time spent in the upstream area, the frequency of upstream area entries, the fatigue duration, and the flow-induced passive drift ratio (Fig. 2D–G). At week 3, no significant differences were observed in the upstream swimming time, entry frequency, or flow-induced passive drift ratio between the 3% NYT and control groups. However, fatigue duration was significantly shorter in the 3% NYT group (*p* < 0.05), indicating improved fatigue resistance. At week 5, 3% NYT-fed fish exhibited increasing trends in upstream swimming time and entry frequency, and significant improvements were observed in both fatigue duration (*p* < 0.05) and flow-induced passive drift ratio (*p* < 0.01). At week 6, 3% NYT treatment resulted in a significant enhancement in three parameters: upstream swimming time, entry frequency, and flow-induced passive drift ratio (*p* < 0.01 for each comparison), and exhibited increasing trends in fatigue resistance. In contrast, zebrafish fed a Gin-supplemented diet for 6 weeks showed no significant differences from the control group in the time spent in the upstream area, the frequency of upstream area entry, or the flow-induced passive drift ratio, although fatigue duration and flow-induced passive drift ratio tended to be reduced (Fig. 2D–G). Furthermore, zebrafish fed the 0.3% NYT diet showed no measurable alterations relative to controls in any of the four parameters (time spent in the upstream area, frequency of upstream area entry, fatigue duration, or flow-induced passive drift ratio), although flow-induced passive drift ratio showed a downward trend (Supplementary Fig. 1). Taken together, these findings indicate that the enhancement of swimming endurance and fatigue resistance under forced swimming conditions was observed specifically in zebrafish fed the 3% NYT diet. Accordingly, based on these results, we selected the 3% NYT diet for all subsequent experiments.

### NYT induces hypertrophy of fast-twitch muscle fibers

**Fig. 3:**
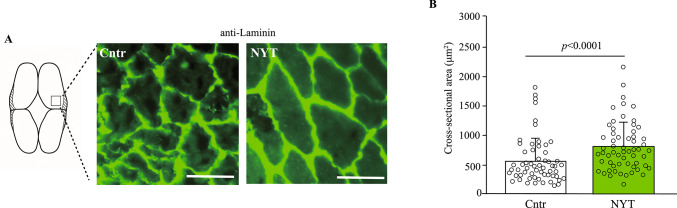
NYT induces hypertrophy of fast-twitch muscle fibers in zebrafish. **A** Representative images of transverse muscle sections stained with anti-laminin antibody. White bar means 50 μm. **B** Quantification of fast muscle fiber cross-sectional area revealed a significant increase in the NYT group compared to the control group. n = 70

To investigate the structural basis of the enhanced swimming performance observed in NYT-fed zebrafish, we performed a histological analysis of the skeletal muscle following six weeks of dietary intervention. Cryosections of the fast-twitch muscle tissue were immunostained with an anti-laminin antibody to visualize the boundaries of individual muscle fibers. Fluorescence microscopy revealed an apparent enlargement of fast-twitch muscle fibers in the NYT-treated group compared to the control group (Fig. 3A). Quantitative analysis of the cross-sectional area showed a significant increase in fiber size in NYT-fed fish, approximately 1.5-fold larger than that in the control group (*p* < 0.0001; Fig. 3B). These results indicate that NYT promotes fast muscle hypertrophy, which likely contributed to the improved endurance and resistance to water flow observed during the forced swimming test.

### NYT modulates gene expression associated with fast-twitch muscle development, differentiation, and metabolism

We analyzed the expression of muscle-related genes in zebrafish following forced swimming to investigate the molecular mechanisms underlying NYT-induced muscle hypertrophy and enhanced swimming performance.

**Fig. 4:**
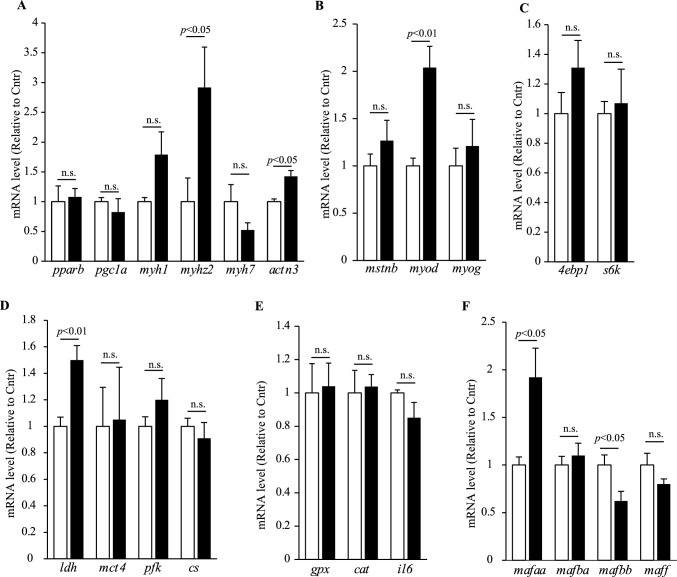
Gene expression analysis of muscle-related pathways in NYT-fed zebrafish. **A** Expression of fast- and slow-twitch muscle fiber genes. **B** Muscle differentiation markers. **C** Genes involved in protein synthesis pathways. **D** Genes associated with energy metabolism. **E** Oxidative stress and inflammatory markers. **F** Expression of Maf family transcription factors. The results are shown as relative levels to Cntr. n = 8–10. n.s., not significant

We first examined the myosin heavy chain isoforms specific to fast-twitch (*myh1* and *myhz2*) and slow-twitch (*myh7*) muscle fibers (Fig. 4A). The expression of *myhz2*, an ortholog of human *MYH4*, was significantly upregulated in the NYT group (*p* < 0.05), and *myh1* expression showed an increasing trend. In contrast, *myh7* expression decreased. No significant changes were observed in *pparb* and *pgc1a* genes, which are involved in slow-twitch muscle maintenance. In addition, *actn3*, a key gene in fast muscle development, was significantly upregulated following NYT treatment (*p* < 0.05). We then evaluated genes involved in muscle differentiation. NYT did not affect the expression of *mstnb*, which encodes myostatin, a negative regulator of muscle growth (Fig. 4B). However, the expression of *myod*, a master regulator of myogenesis, was significantly increased in the NYT group (*p* < 0.01). In contrast, no significant differences were observed in the expression levels of *4ebp1* and *s6k*, which are associated with protein synthesis (Fig. 4C). To explore the role of energy metabolism, we analyzed the genes related to glycolysis and mitochondrial function. Among them, the expression of *ldh*, which encodes lactate dehydrogenase, was significantly elevated in NYT-treated fish after exercise (*p* < 0.01; Fig. 4D), suggesting enhanced anaerobic ATP production. The expression levels of *mct4* (lactate transporter), *pfk,* and *cs* (encoding phosphofructokinase and citrate synthase, respectively) were not significantly altered. We also assessed the markers of oxidative stress and inflammation. No differences were observed in the expression of the antioxidant genes *gpx* and *cat* or the inflammatory cytokine *il6* (Fig. 4E), indicating that these pathways were not major contributors to the observed phenotype. Finally, we investigated the members of the Maf family of transcription factors that have recently been implicated in fast muscle patterning [25]. NYT treatment significantly increased the expression of *mafaa* and decreased *mafbb*, whereas *mafba* and *maff* remained unchanged (Fig. 4F). These findings suggest a potential regulatory role of Maf family members in NYT-induced fast muscle development.

Collectively, these results demonstrate that NYT enhances the expression of genes involved in fast-twitch muscle fiber formation, muscle differentiation, and anaerobic energy metabolism, thereby contributing to improved physical performance in zebrafish.

## Discussion

Maintaining muscle mass and function is critical for preventing sarcopenia and frailty in aging populations. In this study, we demonstrated that dietary supplementation with NYT, a traditional Kampo medicine, significantly enhanced the physical endurance of zebrafish subjected to forced swimming. This improvement was accompanied by hypertrophy of fast-twitch muscle fibers and upregulation of key genes associated with fast muscle differentiation and function.

Histological analysis revealed that NYT treatment increased the cross-sectional area of fast-twitch muscle fibers without affecting food intake, suggesting a direct effect on muscle remodeling. Gene expression profiling indicated that NYT upregulated the expressions of *myhz2* and *actn3*, markers of fast muscle fibers, and *myod*, a master regulator of myogenesis. In contrast, the genes involved in slow-twitch muscle maintenance (*myh7*, *pparb*, and *pgc1a*) and canonical protein synthesis pathways (*4ebp1* and *p70s6k*) were not significantly altered, implying that NYT promotes fast muscle hypertrophy through mechanisms that are distinct from those of the mTOR signaling pathway [26].

NYT increased muscle cell size in a zebrafish model. However, NYT did not affect daily food intake compared with the control group in young adult zebrafish, similar to our previous study using NPY-KO zebrafish [19], indicating that NYT effectively accelerated fast muscle growth. Nevertheless, the mechanism by which NYT enhanced fast muscle growth is still unclear. *myhz2* is a zebrafish ortholog of human MYH4 [27]. Fast-twitch muscle fibers are subdivided into three types: IIa, IIb, and IIx, each with distinct physiological roles and MYH gene expression profiles. MYH4 is specifically expressed in type IIb fibers, which are characterized by rapid contraction and a high glycolytic capacity [25]. Therefore, the more pronounced upregulation of *myhz2* compared to other fast muscle genes, such as *myh1,* following NYT administration suggests that NYT may selectively promote the development of a specific subtype of fast-twitch muscle fibers.

MAF transcription factors are the key regulators of MYH4 expression [25]. In human iPSC-derived myotubes, overexpression of MAFA, MAFB, or MAF enhances the expression of MYH4. Furthermore, human muscle biopsy samples have demonstrated a positive correlation between *MAFA*, not *MAFB,* expression levels and the proportion of fast-twitch fibers. Moreover, the expression of MAFA and MAF, not MAFB, are markedly elevated in individuals engaged in power training compared with those performing endurance training. One intriguing finding was the modulation of Maf family transcription factors in NYT-fed zebrafish. *mafaa* was upregulated and *mafbb* was downregulated in NYT-treated fish, suggesting a possible role for Maf proteins in the promotion of the fast-twitch muscle phenotype. Although the function of the Maf family members in skeletal muscles remains poorly understood, particularly in non-mammalian models, these results suggest that NYT exerts its effects through previously underexplored transcriptional regulators. Further investigations are warranted to elucidate their specific contributions.

In addition to muscle remodeling, NYT enhanced the expression of *ldh*, a key enzyme in anaerobic glycolysis, following forced exercise. This suggests that NYT may augment the energy supply required for high-intensity locomotion, thereby improving resistance to fatigue. The coordination of metabolic adaptations with muscle structural changes indicates a comprehensive enhancement of physical performance.

Previous studies in rodent models have shown that NYT suppresses muscle atrophy by downregulating Atrogin-1 and Murf1, thus highlighting its potential as an anti-sarcopenic agent [12]. Combined with the present findings that NYT can induce muscle hypertrophy under non-atrophic conditions, Kampo medicine may serve as a dual-action therapy that prevents muscle wasting while promoting muscle growth. NYT has also been reported to exert anxiolytic and prosocial effects in zebrafish models with neuropeptide Y deficiency [10]. Given that frailty encompasses physical, psychological, and social dimensions, NYT has emerged as a unique multifunctional agent capable of targeting all three components. The beneficial effects of NYT may stem from synergistic interactions between the 12 constituent herbs. Although the active compound(s) responsible for muscle remodeling remain unknown, several NYT components such as ginseng, Schisandra fruit, and citrus peel have independently demonstrated positive effects on muscle strength, fatigue recovery, and inflammation [28–30]. The polyherbal nature of NYT is consistent with the traditional Kampo philosophy of balancing and amplifying therapeutic effects using multicomponent formulations.

Zebrafish are a valuable model for studying vertebrate muscle development because the molecular programs governing muscle differentiation are highly conserved with those of mammals. The use of zebrafish in this study not only enabled the identification of functional improvements and molecular markers but also provided a platform for future screening of NYT-derived active ingredients. Notably, the observed effects of NYT on muscle performance in zebrafish are consistent with clinical reports of improved vitality and reduced fatigue in elderly humans receiving NYT, thereby supporting its translational relevance.

In conclusion, our findings demonstrate that NYT enhances endurance capacity and promotes fast-twitch muscle hypertrophy through noncanonical molecular mechanisms involving transcriptional regulation and metabolic adaptation. Given that fast-twitch fibers decline with age but can still be increased through exercise training in older individuals [31, 32], the promotion of fast-twitch fiber synthesis by NYT represents a physiologically relevant effect. These data support the potential of NYT as a novel intervention for preventing sarcopenia and frailty, especially in populations with reduced ability or willingness to engage in exercise.

## Supplementary Information

Below is the link to the electronic supplementary material.

**Supplementary Fig. 1** Effects of 0.3% NYT on swimming performance under forced swimming conditions. **A**–**D** Quantitative analysis of swimming performance at weeks 3 and 6. **A** Time spent in the upstream area. **B** Frequency of upstream entries. **C** Fatigue duration. **D** Flow-induced passive drift ratio. n = 10. (PDF 672 kb)

## References

[1] Tsugane S (2021) Why has Japan become the world’s most long-lived country: insights from a food and nutrition perspective. Eur J Clin Nutr 75:921–928. 10.1038/s41430-020-0677-5 32661353 10.1038/s41430-020-0677-5 PMC8189904

[2] Pan N, Ossowski Z, Tong J et al (2024) Effects of exercise on frailty in older people based on ACSM recommendations: a systematic review and meta-analysis of randomized controlled trials. J Clin Med 13:3037. 10.3390/jcm13113037 38892748 10.3390/jcm13113037 PMC11173309

[3] Dyle MC, Ebert SM, Cook DP et al (2014) Systems-based discovery of tomatidine as a natural small molecule inhibitor of skeletal muscle atrophy. J Biol Chem 289:14913–14924. 10.1074/jbc.M114.556241 24719321 10.1074/jbc.M114.556241 PMC4031541

[4] Yu R, Chen J, Xu J et al (2017) Suppression of muscle wasting by the plant‐derived compound ursolic acid in a model of chronic kidney disease. J Cachexia Sarcopenia Muscle 8:327–341. 10.1002/jcsm.12162 27897418 10.1002/jcsm.12162 PMC5377392

[5] Munekawa C, Okamura T, Majima S et al (2024) Daidzein inhibits muscle atrophy by suppressing inflammatory cytokine- and muscle atrophy-related gene expression. Nutrients 16:3084. 10.3390/nu16183084 39339684 10.3390/nu16183084 PMC11434955

[6] Parr M, Botrè F, Naß A et al (2014) Ecdysteroids: a novel class of anabolic agents? Biol Sport 32:169–173. 10.5604/20831862.1144420 10.5604/20831862.1144420 PMC4447764 26060342

[7] Satoh H (2013) Pharmacological characteristics of Kampo medicine as a mixture of constituents and ingredients. J Integr Med 11:11–16. 10.3736/jintegrmed2013003 23464641 10.3736/jintegrmed2013003

[8] Suzuki S, Aihara F, Shibahara M, Sakai K (2019) Safety and effectiveness of Ninjin’yoeito: a utilization study in elderly patients. Front Nutr 6:14. 10.3389/fnut.2019.00014 30873411 10.3389/fnut.2019.00014 PMC6401652

[9] Takayama S, Michihara S, Kimura Y et al (2023) Review of frequently used Kampo prescriptions: part 4, Ninjin’yoeito. Tradit Kampo Med 10:224–252. 10.1002/tkm2.1387

[10] Kawabe M, Hayashi A, Komatsu M et al (2021) Ninjinyoeito improves anxiety behavior in neuropeptide Y deficient zebrafish. Neuropeptides 87:102136. 10.1016/j.npep.2021.102136 33721592 10.1016/j.npep.2021.102136

[11] Kawabe M, Nishida T, Horita C et al (2022) Ninjinyoeito improves social behavior disorder in neuropeptide Y deficient zebrafish. Front Pharmacol 13:905711. 10.3389/fphar.2022.905711 36034826 10.3389/fphar.2022.905711 PMC9411948

[12] Amitani H, Chiba S, Amitani M et al (2022) Impact of Ninjin’yoeito on frailty and short life in klotho-hypomorphic (kl/kl) mice. Front Pharmacol 13:973897. 10.3389/fphar.2022.973897 36353482 10.3389/fphar.2022.973897 PMC9637981

[13] Otsuka S, Matsuzaki R, Kakimoto S et al (2024) Ninjin’yoeito reduces fatigue-like conditions by alleviating inflammation of the brain and skeletal muscles in aging mice. PLoS ONE 19:e0303833. 10.1371/journal.pone.0303833 38768175 10.1371/journal.pone.0303833 PMC11104581

[14] Hudock J, Kenney JW (2024) Aging in zebrafish is associated with reduced locomotor activity and strain dependent changes in bottom dwelling and thigmotaxis. PLoS ONE 19:e0300227. 10.1371/journal.pone.0300227 38696419 10.1371/journal.pone.0300227 PMC11065237

[15] Abou-Dahech MS, Williams FE (2024) Aging, age-related diseases, and the zebrafish model. J Dement Alzheimers Dis 1:48–71. 10.3390/jdad1010004

[16] McClelland GB (2012) Muscle remodeling and the exercise physiology of fish. Exerc Sport Sci Rev 40:165–173. 10.1097/JES.0b013e3182571e2c 22732426 10.1097/JES.0b013e3182571e2c

[17] Palstra AP, Rovira M, Rizo-Roca D et al (2014) Swimming-induced exercise promotes hypertrophy and vascularization of fast skeletal muscle fibres and activation of myogenic and angiogenic transcriptional programs in adult zebrafish. BMC Genomics 15:1136. 10.1186/1471-2164-15-1136 25518849 10.1186/1471-2164-15-1136 PMC4378002

[18] Wen Y, Ushio H (2017) Ferulic acid promotes hypertrophic growth of fast skeletal muscle in zebrafish model. Nutrients 9:1066. 10.3390/nu9101066 28954428 10.3390/nu9101066 PMC5691683

[19] Kawabe M, Nishida T, Takahashi R et al (2023) Comparative study of the effects of the three kinds of Kampo-hozai: Ninjinyoeito, Hochuekkito, and Juzentaihoto on anxious and low sociability behavior using NPY-knockout zebrafish. Front Pharmacol 14:1168229. 10.3389/fphar.2023.1168229 37324500 10.3389/fphar.2023.1168229 PMC10267730

[20] Nishida T, Horita C, Imagawa M et al (2023) Glucosyl hesperidin exhibits more potent anxiolytic activity than hesperidin accompanied by the attenuation of noradrenaline induction in a zebrafish model. Front Pharmacol 14:1213252. 10.3389/fphar.2023.1213252 37663268 10.3389/fphar.2023.1213252 PMC10470464

[21] Ahmad SS, Chun HJ, Ahmad K, Choi I (2024) Therapeutic applications of ginseng for skeletal muscle-related disorder management. J Ginseng Res 48:12–19. 10.1016/j.jgr.2023.06.003 38223826 10.1016/j.jgr.2023.06.003 PMC10785254

[22] Oh G, Men X, La I et al (2025) Fermented red ginseng extract improves sarcopenia-related muscle atrophy in old mice through regulation of muscle protein metabolism. Food Sci Biotechnol 34:793–802. 10.1007/s10068-024-01702-0 39958181 10.1007/s10068-024-01702-0 PMC11822148

[23] Hibarino M, Aoki E, Kubo Y et al (2024) Lactic acid-fermented by-product of Shochu distillery reduces anxiety behavior in Neuropeptide Y knockout zebrafish by the regulation of isotoin neuron. J Mater Cycles Waste Manag 26:3852–3863. 10.1007/s10163-024-02092-5

[24] Gilbert MJH, Zerulla TC, Tierney KB (2014) Zebrafish (*Danio rerio*) as a model for the study of aging and exercise: physical ability and trainability decrease with age. Exp Gerontol 50:106–113. 10.1016/j.exger.2013.11.013 24316042 10.1016/j.exger.2013.11.013

[25] Sadaki S, Tsuji R, Hayashi T et al (2025) Large MAF transcription factors reawaken evolutionarily dormant fast-glycolytic type IIb myofibers in human skeletal muscle. Skelet Muscle 15:19. 10.1186/s13395-025-00391-5 40713776 10.1186/s13395-025-00391-5 PMC12296675

[26] Lang CH, Frost RA, Deshpande N et al (2003) Alcohol impairs leucine-mediated phosphorylation of 4E-BP1, S6K1, eIF4G, and mTOR in skeletal muscle. Am J Physiol Metab 285:E1205–E1215. 10.1152/ajpendo.00177.2003 10.1152/ajpendo.00177.2003 12944322

[27] Qiu J, Fan X, Wang Y et al (2016) Embryonic hematopoiesis in vertebrate somites gives rise to definitive hematopoietic stem cells. J Mol Cell Biol 8:288–301. 10.1093/jmcb/mjw024 27252540 10.1093/jmcb/mjw024 PMC4991667

[28] Cristina-Souza G, Santos-Mariano AC, Lima-Silva AE et al (2022) Panax ginseng supplementation increases muscle recruitment, attenuates perceived effort, and accelerates muscle force recovery after an eccentric-based exercise in athletes. J Strength Cond Res 36:991–997. 10.1519/JSC.0000000000003555 32379240 10.1519/JSC.0000000000003555

[29] Kim K, Ku S, Lee K et al (2018) Muscle-protective effects of Schisandrae Fructus extracts in old mice after chronic forced exercise. J Ethnopharmacol 212:175–187. 10.1016/j.jep.2017.10.022 29107647 10.1016/j.jep.2017.10.022

[30] Ullah A, Sun Q, Li J et al (2024) Bioactive compounds in *Citrus reticulata* peel are potential candidates for alleviating physical fatigue through a triad approach of network pharmacology, molecular docking, and molecular dynamics Modeling. Nutrients 16:1934. 10.3390/nu16121934 38931288 10.3390/nu16121934 PMC11206486

[31] Nilwik R, Snijders T, Leenders M et al (2013) The decline in skeletal muscle mass with aging is mainly attributed to a reduction in type II muscle fiber size. Exp Gerontol 48:492–498. 10.1016/j.exger.2013.02.012 23425621 10.1016/j.exger.2013.02.012

[32] Frontera WR, Meredith CN, O’Reilly KP et al (1988) Strength conditioning in older men: skeletal muscle hypertrophy and improved function. J Appl Physiol 64:1038–1044. 10.1152/jappl.1988.64.3.1038 3366726 10.1152/jappl.1988.64.3.1038

