# Supplementary figures and images for: Ninjin’yoeito Kampo medicine enhances zebrafish endurance under forced-swimming conditions via muscle hypertrophy
Source: J Nat Med. 2026 Apr 21;80(3):709–19. doi: 10.1007/s11418-026-02020-x (PMC13186886; doi:10.1007/s11418-026-02020-x)

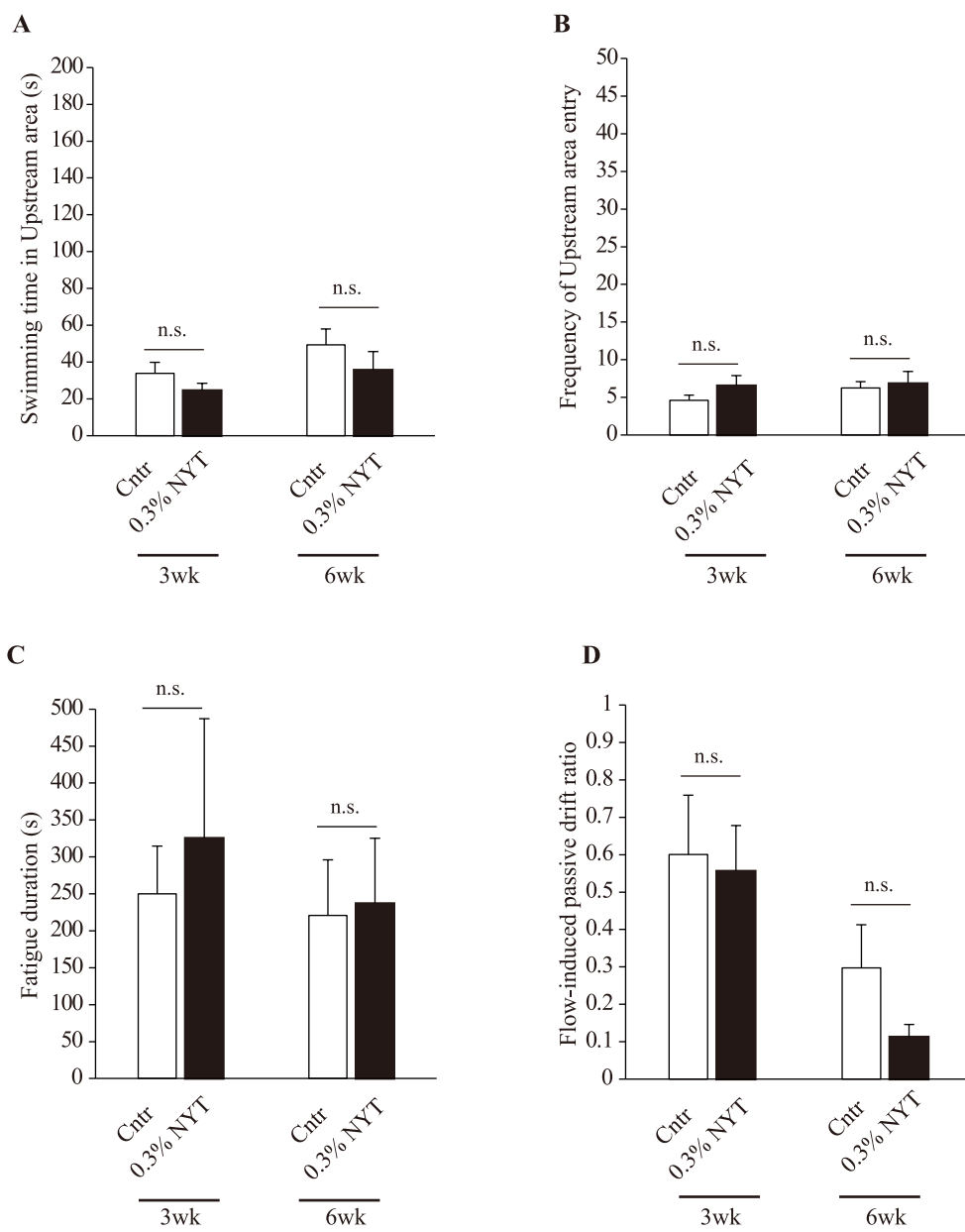

**Supplementary Fig. 1**

Supplement: Supplementary file 1 — Supplementary Fig. 1 Effects of 0.3% NYT on swimming performance under forced swimming conditions. A–D Quantitative analysis of swimming performance at weeks 3 and 6. A Time spent in the upstream area. B Frequency of upstream entries. C Fatigue duration. D Flow-induced passive drift ratio. n = 10. (PDF 672 kb) [file 11418_2026_2020_MOESM1_ESM.pdf]
